# Effect of Ionization Degree of Poly(amidoamine) Dendrimer and 5-Fluorouracil on the Efficiency of Complex Formation—A Theoretical and Experimental Approach

**DOI:** 10.3390/ijms24010819

**Published:** 2023-01-03

**Authors:** Magdalena Szota, Pawel Wolski, Cristina Carucci, Flaminia Cesare Marincola, Jacek Gurgul, Tomasz Panczyk, Andrea Salis, Barbara Jachimska

**Affiliations:** 1 Jerzy Haber Institute of Catalysis and Surface Chemistry Polish Academy of Sciences, 30-239 Krakow, Poland; 2 Department of Chemical and Geological Sciences, University of Cagliari, 09042 Cagliari, Italy

**Keywords:** PAMAM dendrimer, 5-fluorouracil, 5FU, drug delivery systems, DDS, nanotechnology, nanoparticles

## Abstract

Due to their unique structure, poly(amidoamine) (PAMAM) dendrimers can bind active ingredients in two ways: inside the structure or on their surface. The location of drug molecules significantly impacts the kinetics of active substance release and the mechanism of internalization into the cell. This study focuses on the effect of the protonation degree of the G4PAMAM dendrimer and the anticancer drug 5-fluorouracil (5FU) on the efficiency of complex formation. The most favorable conditions for constructing the G4PAMAM-5FU complex are a low degree of protonation of the dendrimer molecule with the drug simultaneously present in a deprotonated form. The fluorine components in the XPS spectra confirm the formation of the stable complex. Through SAXS and DLS methods, a decrease in the dendrimer’s molecular size resulting from protonation changes at alkaline conditions was demonstrated. The gradual closure of the dendrimer structure observed at high pH values makes it difficult for the 5FU molecules to migrate to the interior of the support structure, thereby promoting drug immobilization on the surface. The ^1^H NMR and DOSY spectra indicate that electrostatic interactions determine the complex formation process. Through MD simulations, the localization profile and the number of 5FU molecules forming the complex were visualized on an atomic scale.

## 1. Introduction

Drug delivery methods are being sought in the fight against oncological diseases [1,2]. A wide range of nanoparticles have been exploited as a carrier in drug delivery. Nanocarriers protect anticancer active molecules from biodegradation and provide sustained distribution in the body [1]. While these advances show promise at the preclinical stage, the clinical application of drug delivery nanotechnology is still challenging. To develop drug delivery systems with enhanced treatment efficacy, the physicochemical properties of nanoparticles, such as size, shape, flexibility, surface chemistry, and surface charge, must be precisely determined [2,3,4].

Dendrimers are effectively used for anticancer drug conjugation, encapsulation (drug loading), or complexation (which preserves the chemical integrity and pharmacological drug properties) [5,6,7,8]. In some cases, depending on the structure and nature of the drug–dendrimer interaction, dendrimers can also be associated in clusters when interacting with certain drug molecules [9]. The type of drug–dendrimer interaction at the atomistic level is a key issue in controlled drug delivery science, as it affects the physicochemical properties of the system, its cytotoxicity, and the mechanism and kinetics of drug release, thus the efficacy in vivo. 

Poly(amidoamine) (PAMAM) dendrimers are globular, three-dimensional polymers with symmetric nano-sized structures ranging from 1 to 130 nm [10,11]. PAMAMs are composed of several peripheral groups with positive, negative, or neutral charges. The key features of dendrimers, such as their monodisperse nature and different functional groups on the exposed surface, make them a suitable choice as carriers for targeted drug delivery [6]. Due to their features, they can interact with ligand molecules and enhance their role [5,12]. One of the nanocarriers most widely studied in the literature is the fourth-generation dendrimer (G4PAMAM). This is due mainly to its high drug retention ability, which is usually better than other higher-generation dendrimers (>G6PAMAM) [13,14,15]. Moreover, G4PAMAM dendrimers have an accessible flexible structure compared to the most recent ones [3,16]. The interactions between the dendrimer and the drug consist of various covalent and non-covalent bonds [6,7]. Using 1D and 2D NMR techniques, Gao et al. demonstrated the outermost amino groups of the G5PAMAM dendrimer interacting with 6-mercaptopurine through ionic interactions, with the drug subsequently encapsulated into the inner cavity of the dendrimer through hydrogen, ionic, and hydrophobic bonds [17]. On the other hand, Ybarra et al. investigated the mechanism of interaction between the G4PAMAM dendrimer and Vismodegib using fluorescence emission, FT-IR, DLS, and zeta-potential measurements. They demonstrated the presence of electrostatic interactions between terminal primary amines and nucleophilic clouds of VDG, suggesting mainly surface localization of the drug [18].

In view of the unique properties of dendrimers, they are the subject of numerous reports showing their potential use as carriers for drugs such as cisplatin, methotrexate, doxorubicin, ibuprofen, indomethacin, or 5-fluorouracil [5,6,7,19,20]. Despite a large number of promising in vitro and in vivo studies, very few drugs have managed to advance to clinical trials. Currently, VivaGel^®^, developed by Starpharma Holdings Ltd., is the only commercially available product based on dendrimer drug delivery technology. It contains a sulfonated poly(l-lysine) (PLL) dendrimer [5,6]. Another product developed by the same company is a drug–dendrimer complex called PEGylated PLL-docetaxel conjugate (DEP^®^), which is currently in phase II clinical trials for the treatment of various cancers [6].

The 5-fluorouracil (5FU) drug is a uracil nucleobase analog in which the hydrogen at the 5-position is replaced by fluorine. It has been used as a first-line chemotherapy agent to treat various cancers—mainly colorectal cancer, head, neck, and breast cancer. The general active mechanism occurs via the inhibition of thymidylate synthase, an important chemotherapeutic target with a central position in the DNA biosynthesis pathway [21]. However, due to the limited bioavailability of 5FU, its response rates in advanced colorectal cancer are less than 15% [22,23]. It has a low affinity for biological membranes and its solubility in water increases at pH > 8 (p*K_a_* = 8.0) [23]. In view of these considerations, conventional 5FU therapy requires high concentrations to exploit its drug function, which causes several side effects, including hematologic, nervous, cardiac, and dermatologic reactions and severe gastrointestinal toxicity [21].

The use of PAMAM dendrimers as a drug carrier has been shown to significantly improve the solubility of active substances, including 5FU [24,25,26,27]. Two key parameters determine the biodistribution of the active substance: its solubility and its affinity for cell membranes [23,24]. This solubilization effect is due to the presence of a hydrophobic interior and a hydrophilic surface layer that makes dendrimers behave similar to micelles in solution [28]. Several reports demonstrated that the complexation of lower-generation PAMAM dendrimers with various drugs such as dexamethasone, lidocaine, and doxorubicin results in enhanced permeation compared to the free drug [24]. Indeed, by having a direct effect on drug solubility, PAMAM dendrimer used as a drug carrier can directly affect the interaction between the drug and the site of administration, thus increasing its penetration depth into the body’s tissues. This effect enables optimization of drug concentration, reducing the number of side effects due to high-dose drug therapy. Despite a wide range of research on the delivery of 5FU, an effective system going beyond in vivo and in vitro studies has not yet been developed.

**Figure 1:**
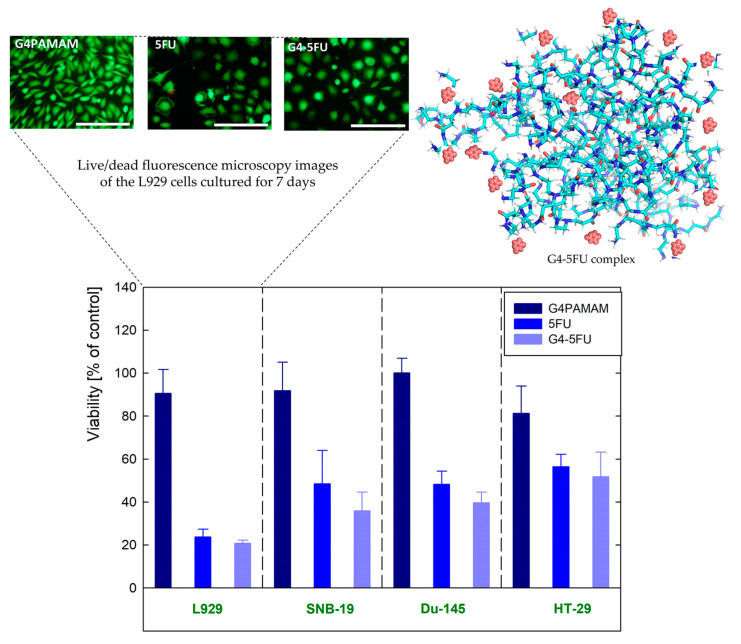
The initial evaluation of cytotoxicity of dendrimers and their complexes with 5FU against mouse fibroblasts (L929), glioblastoma (SNB-19), prostate cancer (Du-145), and colon adenocarcinoma (HT-29) determined via metabolic activity assay of the cells cultured for 7 days in the presence of G4PAMAM dendrimers, G4-5FU complexes, and 5FU, with concentration corresponding to its concentration in complex [15].

Our previous studies, summarized in Figure 1, have shown that under properly optimized conditions, the G4PAMAM dendrimer binds approximately 20 molecules of 5FU, acting as a non-toxic and effective nanocarrier, in agreement with the literature [12,15]. While, so far, attention has been mainly focused on the binding efficiency of 5FU to the PAMAM or PEGylated PAMAM, there is a lack of studies investigating the localization of 5FU in the dendrimer structure. To address this information gap, in the present work, we focused our attention on the type of interactions between G4PAMAM and 5FU at the atomic level. Moreover, we investigated the drug location in the carrier structure (encapsulation, or immobilization on the surface by electrostatic interactions). For this purpose, advanced analytical methods such as Dynamic Light Scattering (DLS), Small Angle X-ray Scattering (SAXS), Nuclear Magnetic Resonance (NMR), and X-ray Photoelectron Spectroscopy (XPS) were used. Finally, we complemented the experimental work with molecular dynamics (MD) simulations for a better characterization of the nature of PAMAM-5FU interactions at the atomic level.

## 2. Results and Discussion

### 2.1. SAXS and DLS

The size of the hydrodynamic diameter of the PAMAM dendrimer is directly related to the degree of protonation of the molecule, which, in turn, is determined by the tertiary and primary amines present in the structure [8]. The primary amino groups on the dendrimer surface have a p*K_a_* of approximately 9.0, while interior tertiary amine groups have a p*K_a_* of 5.8 [29]. Thus, at high pH (8.0–10.0), only the primary amines present on the surface are protonated. As the pH decreases, the tertiary amines inside the structure are gradually protonated. At strong acidic pH, the dendrimer is completely protonated. Electrostatic interactions between protonated functional groups significantly impact the macromolecule’s effect size of the molecule. Consequently, in an acidic environment, the dendrimer structure swells up due to the electrostatic repulsion between primary and tertiary amines, while in an alkaline environment, the dendrimer structure collapses, with electrostatic repulsions being weaker [3,29].

The hydrodynamic radius (*R_H_*) of the dendrimer is determined from the self-diffusion coefficient *D* according to the Stokes-Einstein equation:

(1) $$R_{H}=\frac{k_{B}T}{6\pi \eta D}$$

where *k_B_* is the Boltzmann constant, *T* is the absolute temperature, and *η* is the viscosity of the solution.

**Figure 2:**
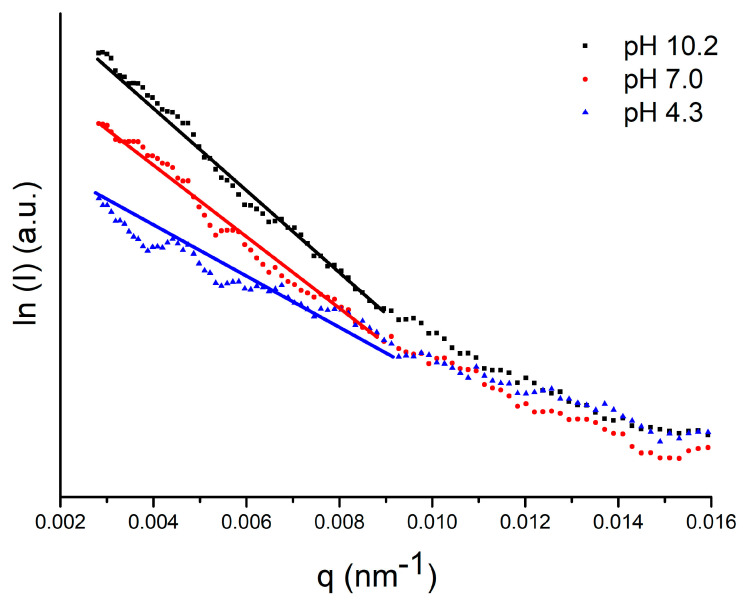
SAXS curves of G4PAMAM dendrimer 2.5% in 1 × 10^−2^ NaCl 10 at different pH.

Small Angle X-ray Scattering (SAXS) was used to investigate the effect of pH on the radius of gyration (*R_g_*) of G4PAMAM-NH_2_. *R_g_* was calculated by using the Guinier model:

(2) $$lnI\left(q\right)\approx -(R_{g}^{2}/3)q^{2}$$

where *q* is the scattering vector (*q* = 0). Because of the exponential nature of the Guinier approximation, *R_g_* can be determined by plotting *ln*(*I*) vs. *q*^2^ (also called the Guinier plot). The region chosen for the linear fit is the Guinier region (Figure 2). The *R_g_* values estimated at pH 10.2, 7.0, and 4.3 are summarized in Table 1.

At pH 10.2, the value of *R_g_* equals 1.87 ± 0.02 nm, which is in good agreement with the literature [30,31,32]. As the pH decreases, and thus the protonation of the dendrimer molecule increases, the value of *R_g_* increases. This trend is similar to Maiti et al., who performed computer simulations on the pH dependence of *R_g_* [33]. The authors estimated an *R_g_* value of 1.68 nm at high pH (>12.0), 1.70 nm at neutral pH (~7.0), and 1.90 nm at low pH (<4.0). A comparison of the *R_g_* values in Table 1 with the hydrodynamic radii (*R_H_*) measured by DLS yielded *R_g_*/*R_H_* ratios of 0.763, 0.790, and 0.777 at pH 10.2, 7.0, and 4.3, respectively, which is consistent with a spherical shape of the molecule (*R_g_*/*R_H_*~0.7–0.8) [34]. As the pH decreases, the *R_H_* and *R_g_* values increase by 13.88% and 16.04%, respectively. This is a consequence of the change in the protonation degree of the molecule. The increase in the protonation degree of the molecule is related to the process of swelling of the dendrimer structure [3,29].

### 2.2. ^1^H NMR Spectroscopy

**Figure 3:**
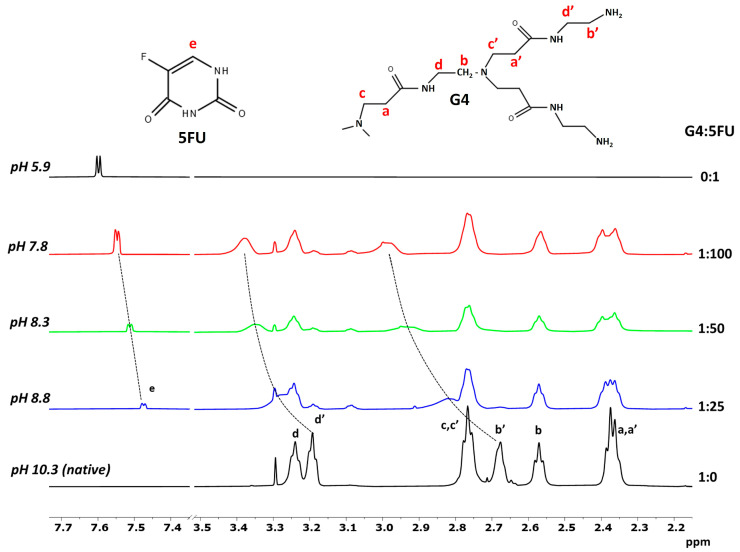
^1^H NMR spectra of G4PAMAM (bottom), 5FU (top), and dendrimer–drug mixtures at different molar ratios and pH (G4PAMAM = 2 mg/mL; 50:50 vol% H_2_O/D_2_O; 30 °C). Labels: a–d denote the methylene protons in the core of dendrimer; a’–d’ denote the methylene proton in the outmost layer of the dendrimer; e denotes the hydrogen occupying position 6 in 5FU. The dotted lines serve to guide the eye only.

The structure of PAMAM dendrimers is characterized by symmetry and repetition of functional groups, making it difficult to distinguish the site of interactions (core and/or shell) with a ligand requiring a range of precise analytical methods [35]. Among these techniques, NMR has been widely used to provide new insights into dendrimer–drug complexes [36]. Indeed, despite the structural complexity of PAMAM and the high number of protons, the ^1^H NMR spectrum of dendrimers exhibits few bands arising from the methylene protons in both the inner and outer layers (Figure 3). Since the chemical shift of a proton can give atomic-level insights into the chemical environment around this nucleus, changes in the chemical shift of the dendrimer and drug signals in the mixture, compared to the chemical shifts found in the individual components, allow the occurrence of host–guest interactions to be revealed. Figure 3 shows the 500 MHz ^1^H NMR spectra of a G4PAMAM solution at pH 10.3, a 5FU solution at pH 5.9, and dendrimer–drug mixtures at G4:5FU molar ratios of 1:25, 1:50, and 1:100 at pH 8.8, 8.3, and 7.8, respectively. As can be seen, the spectrum of dendrimer exhibited six broad bands centered at 2.37, 2.57, 2.68, 2.76, 3.19, and 3.24 ppm. The chemical shift attribution was made based on the literature [37]. Upon the addition of 5FU, discernible proton chemical signals of G4PAMAM were strongly influenced by the presence of the drug. In particular, a progressive downfield shift of the bands at 2.68 and 3.19 ppm, both arising from protons of the outermost layer, occurred at increasing drug concentration. In parallel, the doublet of 5FU also shifted significantly downfield. Furthermore, all dendrimer signals underwent a significant change in line broadening. In good agreement with the literature [37], these NMR findings are considered evidence for the host–guest interaction and, principally, the proximity of the guest molecule to the dendrimer surface, since only the chemical shifts of the terminal methylene protons of G4PAMAM were sensibly affected. This is in good agreement with the results of our previous study, where we demonstrated the effect of 5FU molecules on the zeta potential of the G4PAMAM dendrimer [15]. The higher drug attachment efficiency for the higher dendrimer/5FU molar ratio (1:100) resulted in a reduction in the zeta potential of the system value of approximately 22%. The change in the system’s zeta potential towards a more negative one indicates that the immobilization of the 5FU molecules neutralizes the positive charge from the amino groups present on the surface of the dendrimer molecule [15].

The form in which G4PAMAM and 5FU exist (ionized or neutral) closely determines their behavior and interactions with other systems [38]. The p*K_a_* values for 5FU range between 7.6–8.0. Increasing pH of the environment favors the solubility of 5FU, which is related to the formation of anions in alkaline conditions [39]. Due to the existence of four tautomeric forms of 5FU, the deprotonation process can occur on different atoms. The most stable tautomeric forms are those due to deprotonation from N1 and N3, with the latter being more stable than the former in an aqueous solution [38,40]. Thus, under our experimental conditions, i.e., pH 7.8–10.3, the binding mode is most likely caused by the electrostatic interaction between the protonated surface amine groups of dendrimers and the deprotonated 5FU. This hypothesis is in line with the binding mode of 5FU to G3PAMAM, which has already been discussed in the literature [41]. 

Additional information on the drug–dendrimer system was obtained by DOSY experiments, performed to measure the diffusion coefficient, and thus the hydrodynamic size, of G4PAMAM before and after complex formation. The diffusion coefficient (*D*, m^2^s^−1^) of the dendrimer was determined by fitting the peak-integration decay curves of the DOSY spectra. Compared to the value of *D* for the net dendrimer (8.10 ± 0.01 × 10^−11^ m^2^s^−1^ in 50:50 vol% H_2_O/D_2_O at 30 °C), those measured in the presence of 5FU were lower, progressively decreasing in response to the G4:5FU molar ratio (Table 2). As shown in Table 3, in the presence of 5FU, G4PAMAM displayed hydrodynamic radii higher than the free dendrimer, indicating an increase in the size of the dendrimer. Since the dendrimer internal protons did not give NOE correlations to 5FU (supporting information, Figure S1), suggesting that the binding motif is situated on the surface of the dendrimer, the increase of *R_H_* occurring at the G4:5FU molar ratio of 1:100 (pH 7.8) is likely due to a subsequent protonation of the inner part of the dendrimers, responsible for the swelling of G4PAMAM.

### 2.3. X-ray Photoelectron Spectroscopy (XPS)

**Figure 4:**
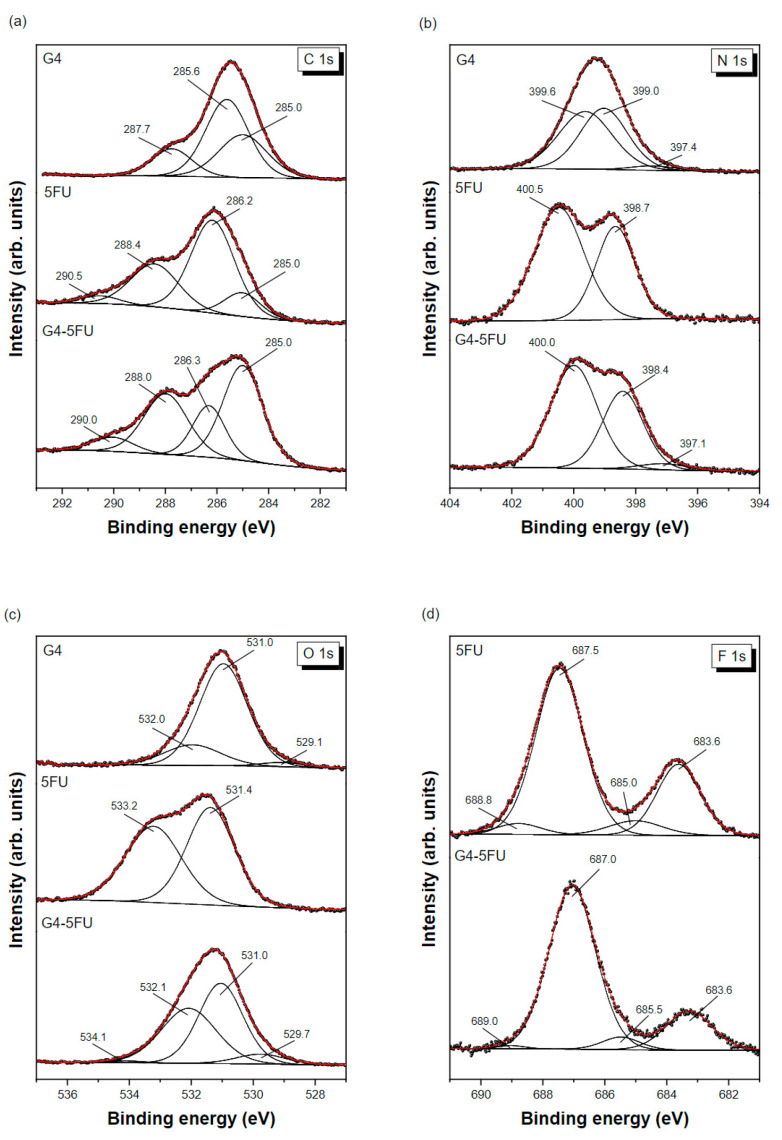
The XPS spectra of G4PAMAM dendrimer, 5-fluorouracil, and G4-5FU layers deposited on Au surface in the BE regions of C 1s (**a**), N 1s (**b**), O 1s (**c**), and F 1s (**d**).

XPS measurements were performed in the binding energy (BE) regions of C 1s, N 1s, O 1s, and F 1s to investigate specific bonds in the dendrimer structure upon interaction with 5-fluorouracil. The experimental C 1s photoemission spectra of G4PAMAM dendrimer, 5FU, and G4-5FU complex deposited on a Au surface are presented in Figure 4a. The binding energies of observed spectral features and components assignments are listed in Table 3. Three components can describe the high-resolution C 1s spectrum of G4PAMAM dendrimer at BE of 285.0, 285.6, and 287.7 eV. These contributions are related to the alkyl C-C/C-H groups, C-O/C-N bonds, and amide carbon in –NH-C=O groups, respectively. The resulting assignment perfectly matches the results showed by Viltres et al. for the fourth generation of poly(amidoamine) dendrimers [42].

Moreover, the lack of a signal near 289.0 eV, which is typical for ester groups, proves the absence of intermediate generations. The C 1s core level spectrum of 5-fluorouracil (Figure 4a) shows four components positioned at BE of 285.0, 286.2, 288.4, and 290.5 eV, which can be assigned to C-C/C-H bonds (including carbon contamination), C-O/C-N (including oxygen contamination), C=O bonds, and C-F bonds, respectively [43]. The slight shift of components towards lower binding energies can be attributed to the interaction of deposited molecules with the Au surface via van der Waals forces, similar to the results reported by Mazolla et al. on 5FU interacting with a silver substrate [44]. The C 1s spectrum of G4-5FU can be fitted by four components’ characteristic for 5-fluorouracil, though, with significantly changed binding energies and peak intensities. This confirms the strong interaction between 5FU and G4PAMAM dendrimer and the formation of the G4-5FU complex.

The N 1s core level spectra of G4PAMAM dendrimer, 5FU, and G4-5FU complex deposited on the Au surface are presented in Figure 4b. In the case of the dendrimer, the spectrum is fitted with three components centered at 397.4, 399.0, and 399.6 eV. The low intensity signal at BE 397.4 eV is from nitrides probably formed during the sample deposition on gold. Much more intense contributions at high binding energies correspond to the unprotonated primary amine (R-C-N) groups (BE = 399.0 eV) and amide (N-C=O) groups (BE = 399.6 eV) belonging to the dendron structure [42]. The relatively high proportion of amides in the total spectrum is related to the fact that the proportion increases with the dendron generation. It is worth noting that the absence of a N 1s signal at BE higher than 401.3 eV indicates there is no protonation of amine groups via local water molecules or the occurrence of inter and intramolecular hydrogen bonds [45]. In line with the literature, 5-fluorouracil is characterized by two peaks in the XPS N 1s spectrum [43,44]. The first component at BE of 398.7 eV can be assigned to the C-NH-C structural group, whereas the second one with BE of 400.5 eV belongs to the N-C=O or N-C-OH groups. There is no signal specific to the protonated amine species C-NH_3_^+^ at 401.7 eV, which could indicate salt formation [43,46]. The N 1s spectrum of G4-5FU complex is similar to that found for 5FU. However, photoelectron peaks shifted to lower binding energies at 398.4 and 400.0 eV. This provides evidence for the interaction between the G4PAMAM dendrimer and 5-fluorouracil and excludes the protonation of nitrogen atoms. Some nitride impurities are also seen at BE of 397.1 eV, in line with the report by Risinggard et al. [47]. 

The O 1s core level spectra of G4PAMAM dendrimer, 5FU, and G4-5FU complex deposited on the Au surface are presented in Figure 4c. The XPS O 1s spectrum of G4PAMAM is characterized by three peaks at 529.1, 531.0, and 532.0 eV. The first small component corresponds to oxygen adsorbed on the gold surface and/or oxygen double bonded with carbon in surface species [43,48]. The other two signals correspond to carboxyl oxygen in the amide groups of PAMAM and C=O bonds. No signal is found above 533.0 eV, where the alkyl O atom of the –O-C=O groups would be found, proving the absence of ester functions in the set of complete dendron generations. It agrees with the G4 C 1s spectrum mentioned above. In the case of 5FU, only two contributions are detected in the XPS O 1s spectrum related to C=O structural bonds (531.4 eV) and OH and/or O-C-O groups (533.2 eV) [43,48]. The G4-5FU spectrum has a much more complicated character and four components are necessary to adequately describe it. Besides the significant contributions characteristic for the dendrimer-5-fluorouracil complex (531.0 and 532.1 eV), there are also two minor contributions coming from oxygen adsorbed on metal (529.7 eV) and chemisorbed water (534.1 eV). It is worth noting that the relative ratio of significant components in the G4-5FU complex is quite different from that found in the parent compounds, which confirms the existence of a strong interaction between them. 

Surprisingly, as many as four contributions have been identified in our F 1s XPS spectrum of 5-fluorouracil (Figure 4d), compared to a single broad component found at 687 eV on monocrystalline surfaces and three components seen on polycrystalline samples [44,47,49]. Two significant peaks most likely correspond to fluorine atoms originating from the structure of 5FU (687.5 eV) and interacting with the gold substrate (683.6 eV). The minor contribution at BE 685.0 eV is probably related to the degradation of 5-fluorouracil, a phenomenon that was reported for silver catalyzed fluorouracil [44,47]. The high BE component at 688.8 eV can be assigned to C-F bonds [50], in agreement with the C 1s spectrum described above. A similar F 1s spectrum was obtained for the G4-5FU complex, with a slightly negative shift in the BE of the highest intensity component. 

### 2.4. Molecular Dynamics (MD)

The experimental research was supported by molecular dynamics simulations. To better understand the effect of protonation degree and complexation process on the dendrimer structure, we quantified the configuration properties using the radii of gyration *R_g,_* three ordered principal moments of inertia *I_x_*, *I_y_*, *I_z_*, and their aspect ratios *I_z_*/*I_x_*, *I_z_*/*I_y_.* In the case of an ideal spherical alignment of dendrimer atoms, all principal moments of inertia would be the same and all aspect ratios would be equal to unity. The asphericity of an object is defined as the deviation of aspect ratios from unity. Thus, an asphericity factor *A_xyz_* was determined for each case according to the definition [51]:

(3) $$A_{xyz}=1-3\frac{\langle I_{2}\rangle }{\langle I_{1}^{2}\rangle }$$

where *I*_1_ and *I*_2_ are defined as $I_{1}=I_{x}+I_{y}+I_{z}$ and $I_{2}=I_{x}I_{y}+I_{y}I_{z}+I_{x}I_{z},\;\mathrm{while}$ 〈…〉 denotes averaging over time. *A_xyz_* quantitatively describes the deviation of an object from spherical symmetry. Its values may vary from 0 (sphere) to 1 (stick). The corresponding values of those parameters, determined for the studied systems, are collected in Table 4.

As seen in Table 4, the protonation degree of the dendrimers has a minimal effect on their shape. Both aspect ratios and asphericity factors are almost the same regardless of the number of protonated amine groups in the dendrimer structure. However, we may tentatively conclude that the S3 system reveals some increase in the gyration radius (size) and the asphericity factor. Electrostatic repulsion between the protonated amine residues likely leads to swelling of the dendrimer and more substantial fluctuations in its shape. This, in turn, leads to the increase of the asphericity factor when compared with the neutral (S1) or less protonated system (S2). 

**Figure 5:**
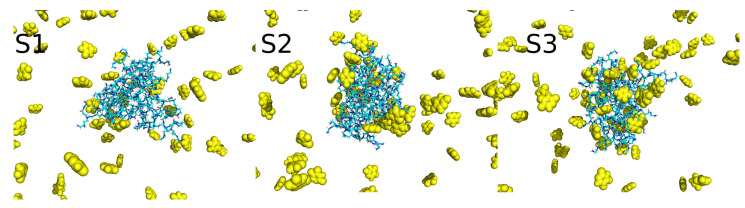
Final structures of the mixed systems: dendrimer (blue) and 100 5FU molecules (yellow).

Figure 5 shows the final configurations of the dendrimer–drug complex composed of the S1, S2, and S3 dendrimers and 5FU molecules after 100 ns of MD simulations. The number of 5FU molecules was the same in each case (100) and, initially, all 5FU molecules were placed at a distance of 5 Å from any atom belonging to the dendrimer structure. 

**Figure 6:**
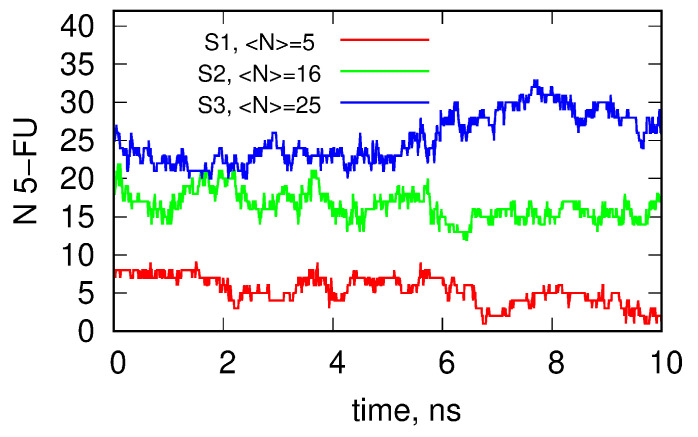
The instantaneous number of 5FU molecules bound to the dendrimer as a function of time obtained in the last 10 ns of simulations.

As can be seen, some 5FU molecules were incorporated into the dendrimer structure, while some 5FU molecules remained in bulk. It is worth noting that Figure 6 shows only one static snapshot of the system structures. The 5FU molecules sometimes dynamically exchange their positions between the dendrimer and the bulk. However, some of the molecules, after entering the dendrimer structure, remain there until the end of the simulation. This is mainly observed in the S3 system. Overall, it seems that the number of 5FU molecules bound within the dendrimer structure increases with the protonation degree of the dendrimer.

To quantify the drug loading capacity of dendrimers at different protonation levels, we calculated the number of 5FU molecules bound within the dendrimer structure. We classify any 5FU molecule as being bound to the dendrimer when the distance between its atoms and any atom belonging to the dendrimer is less than 3.5 Å. Using that definition, we can plot the number of 5FU molecules bound to or encapsulated within the dendrimer as a function of time. Such plots for each protonation degree of the dendrimer are shown in Figure 6.

The average number of 5FU molecules attached to the dendrimer is only 5 for the neutral case S1, grows to 16 for the S2 system, and reaches the maximum value of 25 for the system S3 with the largest number of protonated amine groups. As shown in Figure 6, for S2 and S3 systems, the 5FU molecules are distributed either in the inner or outer space of the dendrimer. In contrast, in the case of the S1 system, the neutral state of the dendrimer, the drug molecules are localized only in outer space. Moreover, the binding of the 5FU molecules is only transient, as they attach briefly to the external atoms of the S1 dendrimer and then detach and move to the bulk. In the case of the S2 and S3 systems, the 5FU molecules also exchange between the dendrimer and the bulk but residence times in the dendrimer are much higher than for the neutral state. 

**Figure 7:**
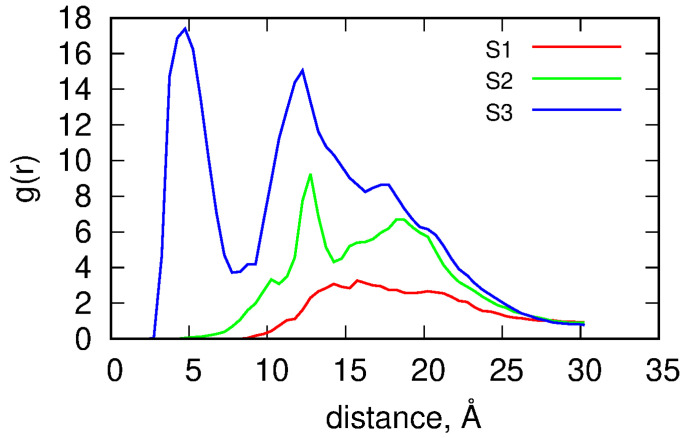
Radial distribution functions determined for the dendrimer–drug molecule distances.

Figure 7 shows the radial distribution functions *g*(*r*) determined for the considered systems. The distances were calculated with respect to the centers of masses of both compounds. Thus, the *g*(*r*) functions give insight into the long-term localization of the 5FU molecules within the dendrimer. Figure 7 shows the qualitative differences between the radial distribution functions for the studied systems.

The S1 system reveals a broad peak with a relatively flat maximum at a distance 15–20 Å from the center of mass of the dendrimer. As shown in Table 4, the dendrimer radius *r* is close to 20 Å since $r=\sqrt{5/3}R_{g.}$ The occupied distances correspond to the attachment of 5FU molecules at the outer space of the dendrimer. At large distances, the probability of finding 5FU molecules decreases and reaches a value for the bulk solution. However, the most crucial observation is that below 15 Å, where there are almost no 5FU molecules, i.e., the drug molecules are localized on the external surface of the dendrimer. In the case of the S2 system, an additional peak appears at a distance of approximately 13 Å. It means that the 5FU molecules are significantly immobilized within the dendrimer structure at such distances. In general, the total number of 5FUs is higher than in the neutral case, but the internal space of the dendrimer is still unoccupied by 5FU molecules. This occupation becomes clear for the S3 system, where a sharp peak appears at a distance of 5 Å. This peak, of course, corresponds to the center of the dendrimer (the distance corresponds to the size of the 5FU molecule) and means that the drug molecules are even more immobilized at this location. The peak at 13 Å for the S3 system is still relatively sharp indicating effective immobilization of drug molecules. The diffusive layer at distances of 15–20 Å is still present and means that some of the 5FU molecules exchange dynamically with the bulk, as observed for the S1 system.

The above conclusions are also supported by the data collected in Table 5. The values *N* (*t* > 10 ns) and *N* (*t* < 10 ns) indicate the numbers of 5FU molecules which remained in the dendrimer structure for more than 10 ns and less than 10 ns, respectively. These numbers were determined within the final simulation window of 10 ns. Thus, it can be seen that in the case of the deprotonated S1 structure, the 5FU molecules attach to the dendrimer structure only transiently and their residence time is short, as none of the molecules remained in the dendrimer for longer than 10 ns. On the other hand, in the S3 system, at least half of the molecules are bound permanently (or for longer than 10 ns) and half of them exchange between bulk and dendrimer space. Finally, the S2 system reveals an intermediate state, as one third of the molecules are bound permanently and two thirds transiently. 

Analysis of the pair interaction energies between the dendrimers and 5FU molecules from Table 4 leads to the conclusion that this energy strongly increases (in absolute values) with the protonation degree of the dendrimer. As can be seen, as the protonation level of dendrimers increases, the electrostatic interactions become the key factor directing the drug to the dendrimers. In the S2 and S3 systems, the electrostatic interactions contribute dominantly to the total interaction energy, resulting in high drug affinity to the carrier. This could be attributed to the strong interaction between positively charged amino groups and the negatively charged drug molecules. By contrast, in the case of the S1 system, where all amine groups are in the non-protonated state, small electrostatic interactions result in an interaction comparable to thermal energy. 

## 3. Materials and Methods

### 3.1. Materials

Poly(amidoamine) dendrimers (PAMAM) generation 4.0 width were acquired from Dendritech, Inc. (Michigan; Midland, MI, USA) and 5-fluorouracil (5FU) and 1,4-dioxane (chromatographic grade, ≥99%) were purchased from Sigma-Aldrich (St. Louis, MO, USA), while deuterium oxide (D_2_O, 99.9 atom% D) was supplied by Deutero GmbH (Kastellaun, Germany).

### 3.2. Dynamic Light Scattering (DLS)

The hydrodynamic radius of the dendrimer was determined by the Dynamic Light Scattering method using a Malvern Nano ZS analyzer. The measurement was performed for a G4PAMAM concentration of 1 mg/mL (Figures S2 and S3).

### 3.3. Small Angle X-ray Scattering (SAXS)

SAXS measurements were performed with S3-MICTRO SWAX camera system (Hecus X-ray System, Graz, Australia), and Cu-K_α_ radiation (λ = 1.542 Å) generated from the Genix X-ray tube, working at 49.9 kV and 0.99 mA. For all measurements, the filters were set to 28,200 and the acquisition time of each curve was 2.5 h. Scattering intensity was registered as a function of scattering vector q = 4πsinθ/λ, where 2θ is the scattering angle and λ is the wavelength of the scattering beam. Samples were placed in glass capillaries (Hilgenberg) of 80 mm length and 2 mm outer diameter. The concentration of dendrimer samples was constant and equal to 2.5 mg mL^−1^. In the scattering curves of samples, the scattering intensity from a capillary filled with 10 mM NaCl was used as a background measurement and subtracted from the scattering intensities. The concentration of the dendrimer sample was constant and equal to 2.5 mg mL^−1^, while the pH was brought to acidic values by adding a few drops of 0.1 M HCl. The value of the radius of gyration (*R_g_*) was calculated using SasView software in the *q* range of 0.00355–0.0636 nm^−1^ at pH = 10.2, 0.00355–0.0547 nm^−1^ at pH = 7.0, and 0.00355–0.0525 nm^−1^ at pH = 4.3.

### 3.4. ^1^H NMR Spectroscopy

A stock solution of G4PAMAM dendrimer (4 mg mL^−1^) was prepared in distilled water. The pure dendrimer sample used for NMR measurements was prepared by diluting 300 μL of the stock solution with D_2_O up to a final concentration of 2 mg mL^−1^ and using 1,4-dioxane (10 μg mL^−1^) as an internal reference. Mixtures of 5FU and G4 were prepared by adding the appropriate amount of 5FU stock solution (4 mg mL^−1^ in D_2_O) to 300 μL of the dendrimer stock solution to produce the desired G4:5FU molar ratios of 1:25, 1:50, and 1:100. The final solutions were diluted with D_2_O up to the final G4 concentration of 2 mg mL^−1^ and a H_2_O:D_2_O ratio of 1:1. Chemical shifts were measured using the proton signal of 1,4-dioxane as an internal reference (0.2 mg mL^−1^). The final pH values of solutions at the G4:5FU molar ratio of 1:0, 1:25, 1:50, and 1:100 were 10.3, 8.8, 8.3, and 7.8, respectively.

^1^H NMR experiments were performed at 300 K on a Bruker Advance III HD 600 spectrometer operating at a 600.13 MHz proton Larmor frequency. One-dimensional ^1^H-NMR spectra were acquired using a standard Nuclear Overhauser Effect Spectroscopy (NOESY) 1Dpresat pulse sequence; noesygppr1d.comp; Bruker BioSpin) with a spectral width of 9600 Hz, an acquisition time of 1.7 s, a relaxation delay of 3 s, a mixing time of 0.01 s, and 8 scans. 

The parameters for 2D NOESY experiment were as follows: mixing time, 200 ms; spectral width, 6000 Hz in both dimensions; acquisition time, 0.142 s; delay time, 2.0 s; number of data points, 2048 (f2) and 256 (f1); number of scans, 8.

BPPLED (Bipolar Pulse Pairs Longitudinal Eddy Current Delay) was performed as the 2D DOSY pulse sequence for each dendrimer–5FU mixture. A 3 s relaxation delay was employed with a total of 8 scans. The diffusion time (Δ) was 300 to 500 ms according to the properties of the samples. The duration of the pulse field gradient (δ/2) was optimized for each diffusion time in the range of 1000–2000 μs to obtain 2–5% residual signal with maximum gradient strength. The delay for gradient recovery was 0.2 ms and the eddy current delay was 5 ms. The pulse gradient was increased from 2% to 95% of maximum strength in a linear ramp (25 steps).

### 3.5. X-ray Photoelectron Spectroscopy (XPS)

X-ray Photoelectron Spectroscopy (XPS) measurements were performed with a hemispherical analyzer (SES R4000, Gammadata Scienta). The unmonochromatized MgKα (1253.6 eV) X-ray source with the anode operating at 12 kV and 15 mA current emission was applied to generate core excitation. The energy resolution of the system, measured as the full width at half maximum (FWHM) for the Ag 3d_5/2_ excitation line, was 0.9 eV (pass energy 100 eV). The spectrometer was calibrated according to ISO 15472:2001. During the experiment, the base pressure in the analysis chamber was approximately 1 × 10^−10^ mbar and approximately 1 × 10^−9^ mbar. 

The sample analysis area was approximately 4 mm^2^ (5 mm × 0.8 mm). All spectra were collected at a pass energy of 100 eV (with 25 meV steps), except survey scans which were collected at a pass energy of 200 eV (with 0.25 eV steps). The XP spectra were registered at an incidence angle of 90° (to the sample surface). Intensities were estimated by calculating the integral of each peak (CasaXPS 2.3.23), after subtraction of the Shirley-type background and fitting the experimental curve with a combination of Gaussian and Lorentzian lines of variable proportions (70:30). 

The samples were weakly conductive; thus, all binding energy values were charge-corrected to the gold Au 4f_7/2_ excitation set at 83.97 eV.

Samples for XPS measurements were prepared by applying a solution of G4PAMAM dendrimer (c = 0.25 mg mL^−1^), 5-fluorouracil (c = 1.84 mg mL^−1^), or G4-5FU complex at a molar ratio of 1:800 onto the Au QCM sensor surface and then evaporated.

### 3.6. MD Simulations—Models and Methods

**Figure 8:**
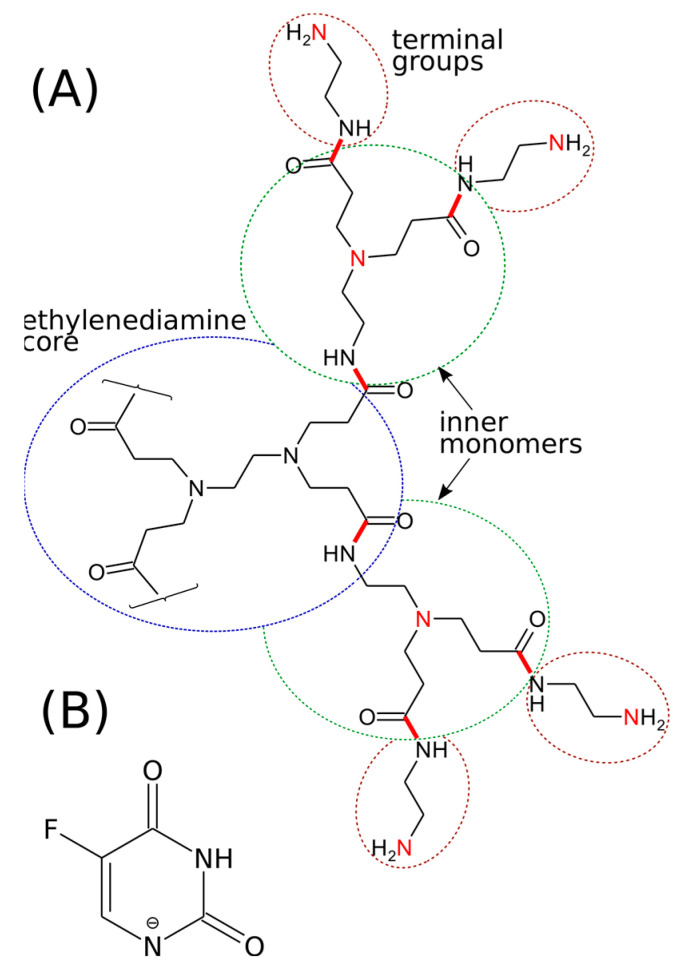
(**A**) Schematic illustration of chemical linkages in the first-generation PAMAM dendrimers. Higher-level generations can be built by repeating inner monomer units. Red sticks denote the linkage between core, inner monomers, and terminal groups, while red N atoms denote nitrogen atoms which can be protonated. (**B**) Chemical structure of the anionic form of the 5-fluorouracil molecule used in simulations.

The three initial structures of the fourth-generation PAMAM dendrimers were produced according to the approach described by Maingi et al. [52]. These three structural templates, denoted as S1, S2, and S3, corresponded to three different protonation levels of PAMAM dendrimers. In system S1, all amine groups are unprotonated, while systems S2 and S3 correspond to dendrimers where 10% and 20% of all amine groups are in the protonated state, respectively. In this way, we were able to track the model’s response to changes in pH. Protonation was assigned randomly to the primary or tertiary amine residues, as is schematically shown in Figure 8A using a fragment of the first-generation dendrimer. The protonation of tertiary amines was justified by either analysis of their role in immobilization of 5FU or by the fact that 5FU leads to a strong reduction of pH of the dendrimer solution, which in turn suggests a strong interaction between these two compounds. Each considered fragment was subjected to geometry optimization using the quantum chemical Hartree–Fock method with a 6–31G(d) basis set. Partial charges were obtained using a restrained electrostatic potential (RESP) procedure [53], implemented in a pyRED [54] program running on a R.E.D. Server Development web page [55]. The total charge of each fragment containing the amine group that underwent protonation was set to +1. Next, using the AcPyPe script [55], the force field and topology files corresponding to the general amber force-field GAFF [56] were generated for each fragment. These files were next used to create atomic structures of the fourth-generation PAMAM dendrimers with given degrees of protonation of amine residues. This final step was conducted using the LEaP program from the AmberTools18 package [57]. The same procedure was applied to the generation of the force-field parameters and atomic partial charges for the studied drug molecule, 5-fluorouracil (5FU, Figure 8B).

To study the effect of the degree of dendrimer protonation on the ability to bind 5FU molecules, we constructed three systems of dendrimer–drug complexes. First, the initial structure of each system was built by placing one of the S1, S2, or S3 dendrimer models in the center of the simulation box, then, 100 5FU molecules in the anionic (deprotonated) form were randomly distributed at a distance of at least 5 Å from the dendrimer (the closest atom belonging to the dendrimer). Using the routines gmx solvate and gmx genion from the Gromacs package (Gromacs is free and open-source software) [58,59], suitable amounts of water TIP3P and Na^+^ ions were then added to the simulation box. Following steepest descents minimization, the equilibration of each system was performed for 200 ps in the NVT ensemble at 300 K. Next, another step of equilibrations was performed in the NPT ensemble, with a reference pressure of 1 bar and temperature of 300 K for 200 ps. Finally, the equilibrated systems were subjected to NPT production runs for 100 ns. A V-rescale thermostat was used to control the temperature with a coupling time of 0.1 ps, while the Parrinello–Rahman barostat controlled the pressure with a coupling constant of 2 ps. Electrostatic interactions were computed using the particle mesh Ewald, PME, method with a 12 Å cutoff in real space. Lennard-Jones interactions were also cut off at a distance of 12 Å. The integration timestep was 2 fs. Hydrogen atoms’ motions were constrained to their equilibrium bond lengths using the LINCS algorithm. The initial box dimension was 120 × 120 × 120 Å^3^. The last 10 ns of the 100 ns production runs were used to collect the results and determine various properties of the system, such as radius of gyration, asphericity parameter, shape factor, radial atomic density distribution, and interaction energies. All simulations were performed using Gromacs. 

## 4. Conclusions

The presented research concerns the effect of the protonation degree of the dendrimer molecule on the formation of a stable complex with 5-fluorouracil. Dendrimers form a stable complex with the drug in the presence of the deprotonated form of 5FU, which has a negative charge. The highest efficiency of the system is observed in conditions of high pH values and at the molar ratio of G4:5FU components equal to 1:100. The formation of the stable complex is mainly attributed to the attractive electrostatic interactions between the positively charged amino groups on the surface of the dendrimer molecule and the negatively charged drug molecules. NMR spectra confirm the drug’s preferred location on the surface rather than the inside. As the pH decreases, the *R_H_* and *R_g_* radii increase by 13.88% and 16.04%, respectively. This is a consequence of the change in the protonation degree of the molecule. The increase in the protonation degree of the molecule is related to the process of swelling of the dendrimer structure. The formed G4-5FU complex is stable after adsorption to the gold surface. The intensity of the component of the XPS spectrum derived from fluorine, contrary to the literature, does not confirm the degradation process of 5FU molecules on the metal surface. Shifts in BE values and changes in the intensity of the XPS spectra components for the G4-5FU complex relative to the initial spectra indicate the existence of strong interactions between the G4PAMAM dendrimer and 5-fluorouracil.

The optimal conditions for complex formation obtained from the experimental work were applied in molecular dynamics simulations. The degree of protonation of the dendrimer molecule was set in the range of 0–20%, corresponding to the conditions in the system at high pH values. MD simulations confirmed the critical influence of electrostatic forces on forming a stable complex. The correlations observed in simulations indicate an increase in the number of 5FU molecules associated with a dendrimer with an increase in the degree of their ionization. For the highest degree of protonation tested, 20% protonation, the average number of 5FU molecules bound to the dendrimer is approximately 25, which agrees with the experimental results. Moreover, the protonation degree also influences the average residence time of the drug molecules in the dendrimer structure. At 20% protonation, half of the 5FU molecules bound to the dendrimer are permanently immobilized, while the other half is dynamically mass exchanged. The lower degree of protonation reduces the average number of drug molecules attached and the number of stably immobilized 5FU molecules. The main conclusion drawn from these studies is that even a small amount of protonated amino groups leads to a significant increase in the number of 5FU molecules bound to the dendrimer. On the other hand, too high a degree of protonation of the dendrimer structure leads to the stiffening of the structure of the molecule, thus blocking the entry of the drug into the structure.

## Figures and Tables

**Table 1:** Average values of the radius of gyration *R_g_* acquired by ^[a]^ SAXS and ^[b]^ MD for G4PAMAM dendrimer. Values of the hydrodynamic radius *R_H_* were determined from ^[c]^ DLS measurements. (*I* = 1 × 10^−2^ NaCl).

pH	^[a]^ *R_g_* (nm)	^[b]^ *R_g_* (nm)	^[c]^ *R_H_* (nm)
	SAXS	MD	DLS
10.2	1.87 ± 0.02	1.47 ± 0.01	2.45 ± 0.05 [12]
7.0	2.11 ± 0.02	-	2.67 ± 0.05
4.3	2.17 ± 0.02	-	2.79 ± 0.05

**Table 2:** The self-diffusion coefficients (*D*) and hydrodynamic radius (*R_H_*) values of G4PAMAM at different G4:5FU ratios (G4PAMAM = 2 mg/mL; 50:50 vol% H_2_O/D_2_O, 30 °C) ^a^.

G4:5FU	pH	*D* (m^2^/s) × 10^−11^	*R_H_* (nm)
1:0	10.3	8.10 ± 0.07	2.97 ± 0.03
1:25	8.8	7.96 ± 0.07	3.03 ± 0.03
1:50	8.3	7.73 ± 0.13	3.12 ± 0.06
1:100	7.8	6.44 ± 0.18	3.7 ± 0.1

^a^ Mean value ± standard deviation (*n* = 3).

**Table 3:** XPS data of G4PAMAM, 5FU, and G4-5FU complex.

Core Excitation	G4 BE (eV) Area (%)	5FU BE (eV) Area (%)	G4-5FU BE (eV) Area (%)	Assignation
O 1s	529.1 2.9	---	529.7 6.8	O–metal(support)
	531.0 78.2	531.4 52.1	531.0 49.8	carboxyl in PAMAM amide group
	532.0 18.9	533.2 47.9	532.1 42.5	C=O/OH
	---	---	534.1 0.9	chemisorbed water
N 1s	397.4 4.1	---	397.1 3.5	=N–
	399.0 59.7	398.7 39.1	398.4 38.1	C–NH–C
	399.6 36.2	400.5 60.9	400.0 58.4	N–C=O
C 1s	285.0 31.9	285.0 11.9	285.0 43.6	C–C/C–H
	285.6 51.1	286.2 55.8	286.3 19.1	C–O/C–N
	287.7 17.0	288.4 28.3	288.0 30.6	–NH–C=O
	---	290.5 4.0	290.0 6.7	C–F
F 1s	---	683.6 25.1	683.6 18.0	F–metal (support)
		685.0 6.2	685.5 4.8	F in degraded 5FU
		687.5 64.7	687.0 76.3	F in 5FU
		688.8 4.0	689.0 0.9	C–F

**Table 4:** Mean values of the radii of gyration *R_g_*, aspect ratio, and asphericity factor for G4PAMAM dendrimers at various protonation degrees.

	Protonation Degree (%)	*R_g_* (nm)	Aspect Ratio		Asphericity Factor
			*I_z_/I_x_*	*I_z_/I_y_*	
S1	0	1.47 ± 0.01	1.50 ± 0.11	1.20 ± 0.03	0.0136
S2	10	1.46 ± 0.01	1.60 ± 0.05	1.12 ± 0.02	0.0178
S3	20	1.54 ± 0.02	1.73 ± 0.07	1.19 ± 0.04	0.0238

**Table 5:** Average pair interaction energies between 5FU molecules (LJ—Lennard-Jones contributions, Coul—electrostatic contributions) and the dendrimer and the number of 5FU molecules remaining in the dendrimer structure for more (less) than 10 ns.

Property/System	S1			S2			S3		
Energy (kJ/mol) per single 5FU molecule	Total	LJ	Coul	Total	LJ	Coul	Total	LJ	Coul
	−4.60	−2.74	−1.83	−21.47	−7.93	−13.54	−34.50	−12.10	−22.40
*N*(*t* > 10 ns)	0			6			12		
*N*(*t* < 10 ns)	8			12			13		

## Data Availability

Not applicable.

## Supplementary Materials

The supporting information can be downloaded at: https://www.mdpi.com/article/10.3390/ijms24010819/s1.

## Abbreviations

PAMAM dendrimers	poly(amidoamine) dendrimers
G4PAMAM	poly(amidoamine) dendrimer of fourth generation
5FU	5-Fluorouracil
G4PAMAM–5FU	5-Fluorouracil complex with fourth generation of dendrimer
R_g_	gyration radius
R_H_	hydrodynamic radius
D	self-diffusion coefficient
BE	binding energy
