# Supplementary material for: Effect of Ionization Degree of Poly(amidoamine) Dendrimer and 5-Fluorouracil on the Efficiency of Complex Formation—A Theoretical and Experimental Approach
Source: Int J Mol Sci. 2023 Jan 3;24(1):819. doi: 10.3390/ijms24010819 (PMC9821593; doi:10.3390/ijms24010819)
Supplement: Supplementary file 1 [file ijms-24-00819-s001.zip › ijms-2108261-supplementary.pdf]

Supplementary material

Effect of ionization degree of poly(amidoamine) dendrimer and 5-fluorouracil on the efficiency of complex formation- theoretical and experimental approach

Magdalena Szota[1], Paweł Wolski[1], Cristina Carucci[2], Flaminia Cesare Marincola[2], Jacek Gurgul[1], Tomasz Panczyk[1], Andrea Salis[2], Barbara Jachimska[1*]

[1]Jerzy Haber Institute of Catalysis and Surface Chemistry Polish Academy of Sciences, Krakow, Poland
[2] Department of Chemical and Geological Sciences, University of Cagliari, 09042 Monserrato (CA), Italy

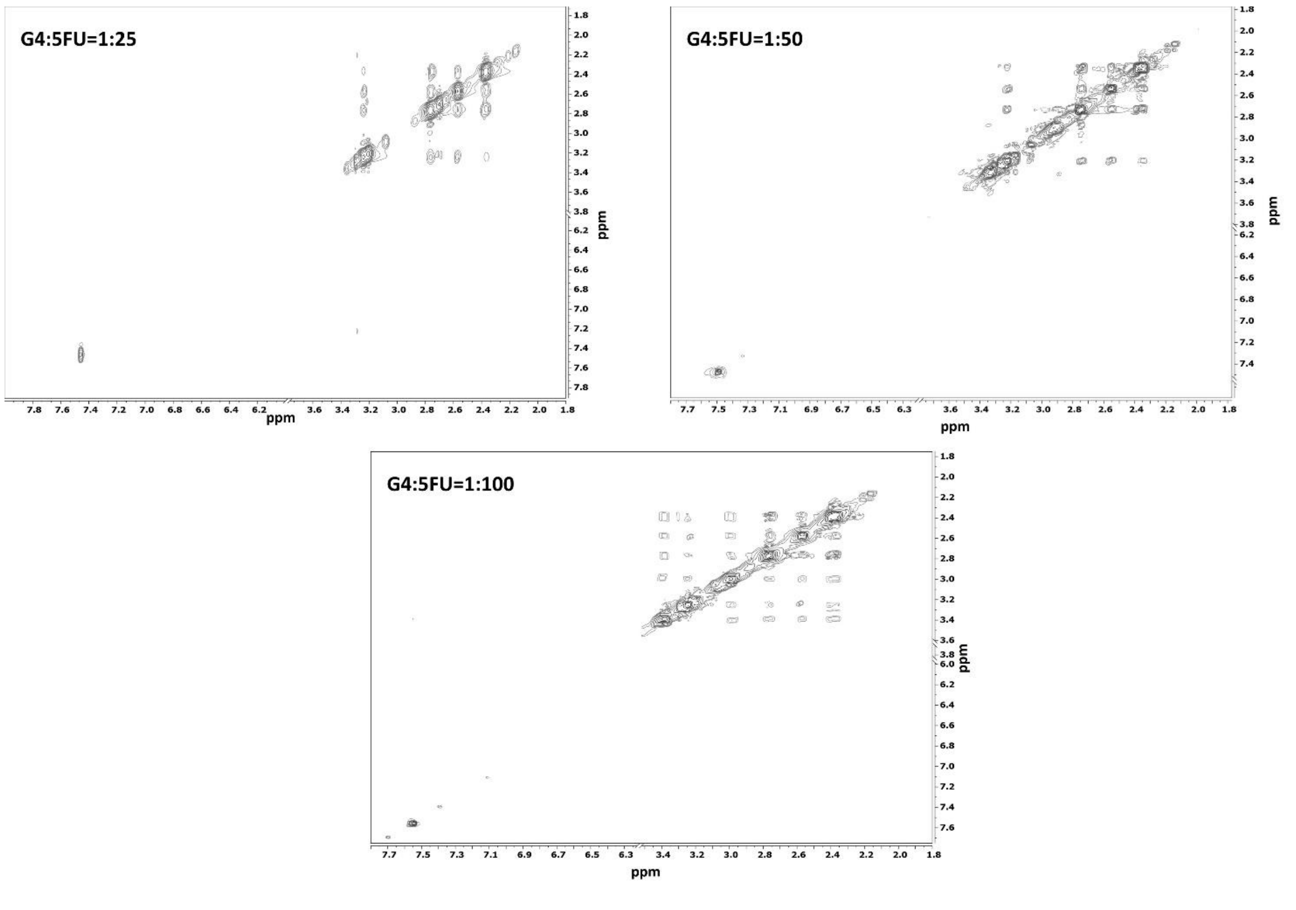

Figure S1. Two dimensional (2D) NOESY NMR spectra for G4 PAMAM dendrimer and 5FU mixture (G4= 2 mg/ml; 50% H_2O/50% D_2O; 30 °C).

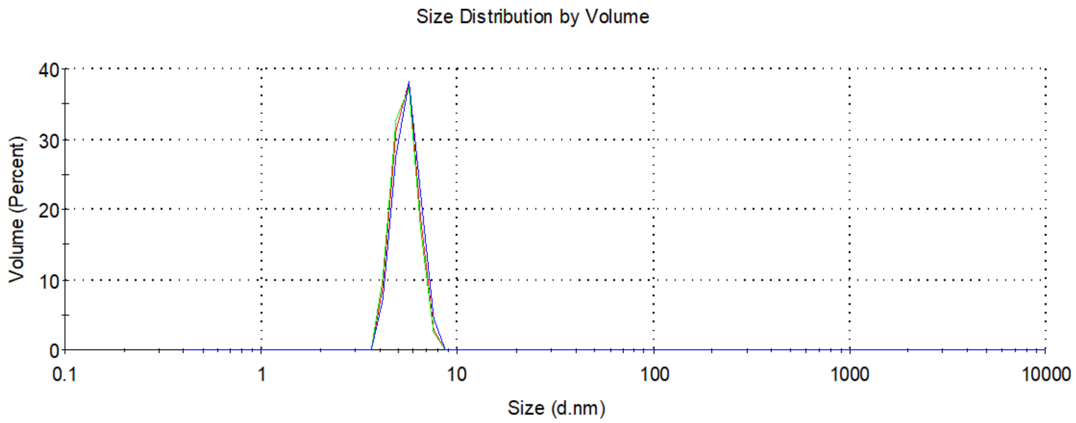

Figure S2 Particle size distribution by volume for G4 pH 7.5.

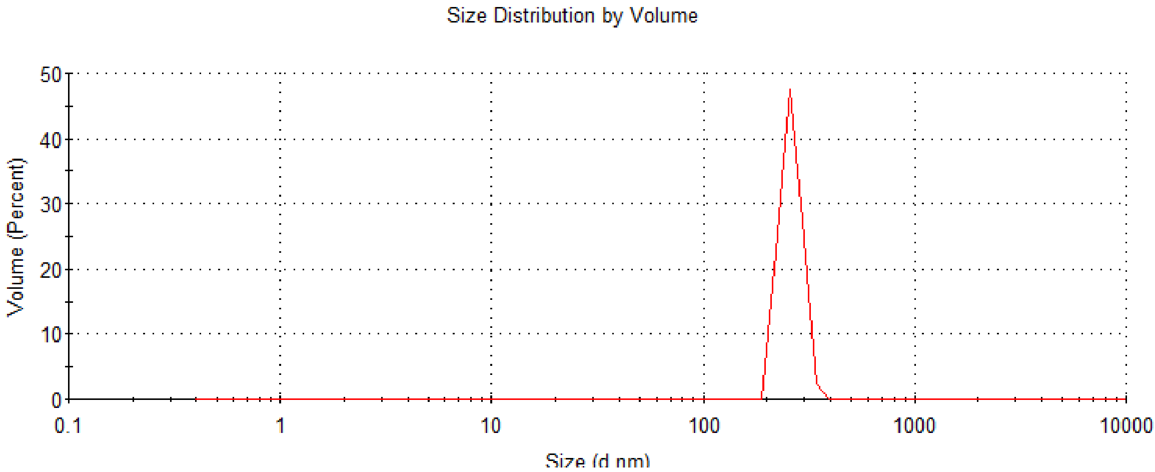

Figure S3. Particle size distribution by volume for G4-5FU complex after dialysis pH 7.5.
